# ATP promotes immunosuppressive capacities of mesenchymal stromal cells by enhancing the expression of indoleamine dioxygenase

**DOI:** 10.1002/iid3.236

**Published:** 2018-10-10

**Authors:** Ramin Lotfi, Lena Steppe, Regina Hang, Markus Rojewski, Marina Massold, Bernd Jahrsdörfer, Hubert Schrezenmeier

**Affiliations:** ^1^ Institute for Transfusion Medicine University of Ulm Ulm Germany; ^2^ Institute for Clinical Transfusion Medicine and Immunogenetics Ulm German Red Cross Blood Services Baden‐Wuerttemberg‐Hessen Ulm Germany

**Keywords:** cancer, DAMPs, inflammation, mesenchymal stromal cells, wound healing

## Abstract

**Introduction:**

MSCs are often found within tumors, promote cancer progression and enhance metastasis. MSCs can act as immuosuppressive cells, partially due to the expression of the enzyme indoleamine dioxygenase (IDO) which converts tryptophan to kynurenine. Decreased concentration of tryptophan and increased kynurenine, both interfere with effective immune response. Damage associated molecular patterns (DAMPs) including ATP are found within the tumor microenvironment, attract MSCs, and influence their biology.

**Methods:**

Bone marrow derived MSCs were exposed to ATP for 4 days, in the presence of 100 ng IFNγ/mL. Intracellular expression of IDO in MSCs was assessed by FACS. Conditioned media from thus stimulated MSCs was analyzed for kynurenine content and its suppressive effect on lymphocyte proliferation. Apyrase or P2 × 7‐receptor antagonist (AZ 11645373) were applied in order to inhibit ATP induced effect on MSCs.

**Results:**

We demonstrate, that ATP at concentrations between 0.062 and 0.5 mM increases dose dependently the expression of IDO in MSCs with subsequent increased kynurenine concentrations within the supernatant at about 60%. This effect could be abolished completely in the presence of ATP degrading enzyme (apyrase) or when MSCs were pretreated with a P2 × 7‐receptor antagonist (AZ 11645373). Consistently, supernatants from MSCs stimulated with ATP, inhibited lymphocyte proliferation from 65% to 16%.

**Conclusions:**

We characterized ATP as a DAMP family member responsible for necrosis‐induced immunomodulation. Given the increased concentration of DAMPs within tumor tissue and the fact that DAMPs can act as chemotattractants to MSCs, our results have implications for therapeutic strategies targeting the tumor microenvironment.

## 1 INTRODUCTION

Regardless of the origin and the site of neoplastic cells, necrotic cell death is a characteristic feature associated with advanced solid tumors. This is likely due to three primary causes: 1) inadequate nutrient supply to tumor cells as a consequence of imbalance between tumor growth and angiogenesis; 2) a cytotoxic immune response to apoptosis‐resistant tumors; and 3) tumor's primary deficiencies in programmed (apoptotic) cell death due to defects in p53 associated pathways, upregulation of BCL2 anti‐apoptotic proteins, and exaggerated autophagy “programmed cell survival.”

Factors released following stress and/or necrotic cell death are also referred to as damage associated molecular patterns (DAMPs). These factors act as “danger” signals recruiting inflammatory cells, inducing inflammation (cytotoxic environment) on the one hand, but promoting wound healing processes (immunosuppressive environment), on the other. While wound healing is itself a self‐limiting process, advanced solid tumors—being “addicted to death”^1^—perpetually release DAMPs which promote recruitment of MSCs,^2^ enhance angiogenesis, and limit immunity.^3^ Thus, the tumor coopts the regenerative capacity of the host^1^, ^4^ resulting in tumor's own survival and proliferation. MSCs can migrate to the sites of inflammation and damage^5^ within the tumor microenvironment.^6^, ^7^, ^8^, ^9^, ^10^ Consistently, MSCs are often found within tumors^11^ and promote cancer progression and enhance metastasis.^9^, ^12^ Systemically transferred MSCs, for example, can also migrate into tumor tissue such as colon carcinomas or glioblastoma.^13^, ^14^ MSCs can act as immunoregulatory cells, releasing IL‐10 and prostaglandin E2, or by expressing the enzyme indoleamine 2,3‐dioxygenase (IDO).^15^, ^16^, ^17^

IDO converts the essential amino acid tryptophan to kynurenine. Local decrease of tryptophan and increase of kynurenine is associated with immunosuppression and a shift from a Th1 to a Th2 response.^18^ IDO expression in MSCs is induced by IFNγ which is commonly generated within inflammatory tissue.^16^

Identified DAMPs include S100 proteins, uric acid, high mobility group box 1 (HMGB1) protein, hyaluronan, heat shock proteins, heparan, syndecan, versican, and adenosine 5′‐triphosphate (ATP).^19^

ATP possesses high metabolic lability, especially at the beta‐gamma and alpha‐beta phosphodiester bonds, which allows its intracellular pools (steady state concentrations) to fluctuate in response to extracellular conditions that affect growth or cellular metabolism.

The baseline blood plasma ATP is between 0.116 and 0.072 μM.^20^, ^21^ Haskell et al reported a range of 0.56–1.49 mM (mean ± 2 standard deviations) in total blood ATP pools at baseline among hospitalized patients without cancer and eight patients with advanced cancer.^22^ ATP at concentrations between 1 and 5 μM have been reported to be cytotoxic to many tumor cells.^23^ Extracellular ATP cytotoxicity was shown to involve permeabilization or pore formation of tumor cell membrane leading to tumor cell death,^24^ a condition which induces intracellular ATP release from necrotic tumor, itself. However, ATP is also released by mature erythrocytes^25^, ^26^, ^27^ whenever they are affected by hypoxia and deformation.^28^, ^29^ Developing tumors are notorious for their compressed tumor vessels producing deformed erythrocytes due to solid stress and a hypoxic microenvironment.^30^ The stress in the tumor microenvironment is also a result of interstitial fluid accumulation. The sudden decrease in oxygen tension in the vicinity of the developing tumor along with the severe impairment of perfusion pressure in the tumor are expected to serve as a stimulus for the increases in release of erythrocyte ATP pools into the blood plasma in a stimulus‐secretion manner. Thus, about thousand times higher ATP concentrations (e.g., 100 μM) are found within tumor microenvironment when compared to normal tissue (10–100 nM).^31^, ^32^, ^33^

Based on the described observations, we aimed to study the influenced of ATP on immunoregulatory capacities of MSCs, in order to better characterize the immunosuppressive milieu of tumor to pave the way for a possible treatment strategy targeting tumor's microenvironment.

## 2 MATERIALS AND METHODS

### 2.1 Ethics statement

This study was conducted according to the principles expressed in the Declaration of Helsinki. The study was approved by the Institutional Review Board (Ethical Committee) of the University of Ulm. All bone marrow and blood donors provided written informed consent for the collection of samples for the generation of MSCs and the isolation of peripheral blood mononuclear cells (PBMCs), as well as for subsequent analyses.

### 2.2 Standard cell culture conditions and used cells

As standard cell culture medium (if not otherwise indicated) phenol‐red free DMEM (Gibco, Schwerte, Germany) supplemented with 10% human serum of blood group AB (German Red Cross Blood Services Baden‐Württemberg‐Hessen) was used. All cells were incubated in a humidified atmosphere at 37°C with 5% CO_2_.

The colorectal tumor cell line HCT‐116 was purchased from the German Collection of Microorganisms and Cell Cultures (DSMZ, Braunschweig, Germany).

### 2.3 Isolation and characterization of bone marrow derived human MSCs

Heparinized bone marrow was taken from healthy volunteers and cultured in alpha‐modified minimal essential medium (α‐MEM, Gibco) supplemented with 10% human platelet lysate (PL from German Red Cross Blood Services Baden‐Württemberg‐Hessen) and containing 100 U/mL penicillin, and 100 μg/mL streptomycin (Gibco). Adherent cells at a confluency below 80% were harvested and passaged following trypsinization. Only MSCs from passage 1 to 3 were used. MSCs were characterized based on surface antigen expression analyzed by flow cytometry using a FACScan flow cytometer with CellQuest 3.1 software (Becton‐Dickinson, Heidelberg, Germany). MSCs (1 × 10^5^ cells) were stained in PBS for 15 min at room temperature using the following antibodies according to the manufacturer's recommendations:

CD105 FITC (clone SN6; AbD Serotec, Düsseldorf, Germany), CD3 PerCP (clone SK7), CD34 PE (clone 8G12), CD45 PerCP (clone 2D1), CD73 PE (clone AD2), CD90 FITC (clone 5E10), (HLA‐DR, DP, DQ) FITC (clone TÜ39), (HLA‐A, B, C) PE (clone G46‐2.6), IgG (clone X40) conjugated with FITC, PE, or PerCP, respectively (all from Becton‐Dickinson). Bone marrow‐derived adherent cells which expressed CD73, CD90, CD105, and Class I molecules (HLA‐A, B, C) and were negative for expression of CD3, CD34, CD45, and Class II molecules (HLA‐DR, DP, DQ) were defined as MSCs. Experiments were performed only on MSCs which were cultured for at least 3 days in standard cell culture media as described above.

### 2.4 Flow cytometric assessment of intracellular IDO in MSCs

MSCs were fixed and permeabilized with Cytofix/Cytoperm (Becton‐Dickinson). In the presence of 10 μg/mL mouse whole IgG (Jackson ImmunoResearch), fixed and permeabilized MSCs were stained with murine anti‐human IDO (Alexa Fluor 488) purchased from R&D systems. FACScan flow cytometer with CellQuest 3.1 software (Becton‐Dickinson) were used to assess intracellular expression of IDO.

### 2.5 Isolation and assessing proliferation of peripheral blood lymphocytes (PBLs)

PBLs were purified from healthy donors’ whole blood by density gradient centrifugation using Biocoll (Biochrom AG, Germany) followed by separation from monocytes based on plastic adherence. Following staining with Carboxyfluorescein succinimidyl ester (CFSE Celltrace, Life Technologies) according to manufacturer's instruction, PBL (10^5^ cells/200 μL) were incubated for 4 days in the presence of anti‐CD3/anti‐CD28 beads (Gibco, Thermo Fischer Scientific, Schwerte, Germany) in conditioned media from MSCs. FACScan flow cytometer with CellQuest 3.1 software (Becton‐Dickinson) was used to assess proliferation of PBL.

### 2.6 Preparation of conditioned media from MSCs

MSCs were cultured in standard culturing media (see above) saturated with 100 μg tryptophan/mL media. Following 4 days of stimulation with ATP at indicated concentrations in the presence of 100 μg IFNγ/mL, the supernatant was obtained and used as conditioned media while the MSCs were fixed, permeabilized, and stained for intracellular IDO.

### 2.7 Assessing remaining ATP in conditioned media from MSCs

Conditioned media from ATP‐stimulated MSCs was tested for remaining ATP by using Luminescent ATP Detection Assay Kit (Abcam, Berlin, Germany) following manufacturer's instructions. Samples on 96 well plates were measured on POLARstar Omega plate reader (BMG Labtech, Germany) and the data were analyzed by MARS Omega data analysis software (BMG Labtech).

### 2.8 Kynurenine measurement

Samples (60 μL) were pipetted in a 96 well‐plate with U‐shaped bottom, 30 μL of 30% trichloroacetic acid (TCA) was added to each well. After an incubation step (30 min) at 50°C in the waterbath, the plate was allowed to cool down while preparing Ehrlich reagent consisting of 2 % p‐Dimethylaminobenzaldehyd in glacial acetic acid (0.2 g powder + 10 mL acetic acid). One part freshly prepared Ehrlich reagent (90 μL) was added to one part samples/standards + TCA in each well. Following 5 min of incubation at room temperature, the plate was centrifuged for 5 min at 3220*g*. About 120 μL of the supernatants was transferred to a 96 well‐plate with flat bottom. Absorbance was measured at 492 nm using POLARstar Omega (BMG Labtech) plate reader. The data were analyzed by MARS (Omega‐data analysis software, BMG Labtech).

### 2.9 Statistics

Student's *t*‐test for means (paired two samples) was used for calculating significance, and *P* values equal or below 0.05 were considered as significant.

## 3 RESULTS

### 3.1 ATP enhances IFNγ‐induced kynurenine production in MSCs

**Figure 1:**
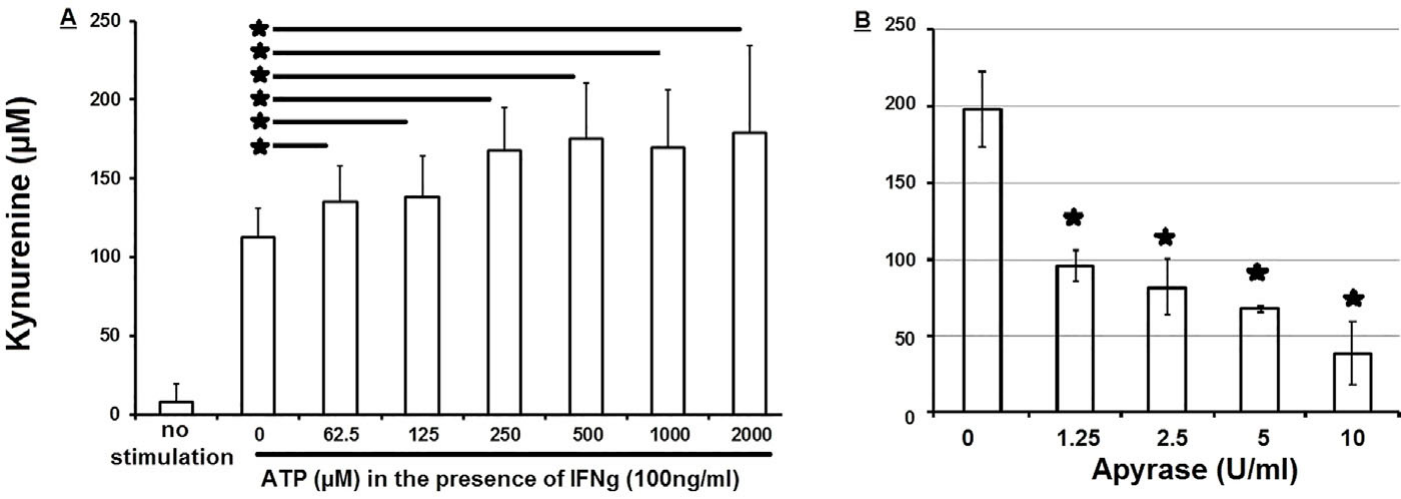
ATP dose‐dependently enhances kynurenine production by MSCs. (A) In the presence of 100 ng IFNγ/mL culturing media containing also 100 μg tryptophan/mL, bone marrow derived MSCs (15 × 10^3^ cells/cm^2^) were stimulated with indicated concentrations of ATP for 4 days and kynurenine concentration was assessed within the supernatant by a colorimetric assay. Shown is one out of eight independent experiments with mean values and error bars indicating standard deviation. (B) In the presence of indicated concentration of ATP‐degrading enzyme apyrase in culturing media supplemented with 100 μg tryptophan/mL, bone marrow derived MSCs (15 × 10^3^ cells/cm^2^) were stimulated for 4 days with IFNγ (100 ng/mL) and ATP (500 μM). Apyrase dose‐dependently inhibits ATP‐enhanced kynurenine production in MSCs. Shown are representative results (mean ± standard deviation) from one out of four individual experiments

ATP is released following inflammation, hypoxia, tissue injury, and necrotic cell death which are conditions associated with recruitment of MSCs. IFNγ is an inflammation‐associated cytokine known to induce IDO expression in MSCs, thus we stimulated bone marrow derived MSCs with graded concentrations of ATP in the presence of 100 ng IFNγ/mL and could demonstrate a dose dependent increase in kynurenine concentrations in supernatants from MSCs after 4 days of incubation, when compare to non‐stimulated MSC or MSCs stimulated with IFN alone (Figure 1A). The optimal ATP concentrations enhancing kynurenine production ranged between 250 and 500 μM (Figure 1A). To demonstrate the specificity of ATP effect on MSCs, we repeated the experiments using 500 μM ATP in the presence of graded concentration of ATP‐degrading enzyme apyrase abolishing the stimulatory effect of ATP on MSCs (Figure 1B).

**Figure 2:**
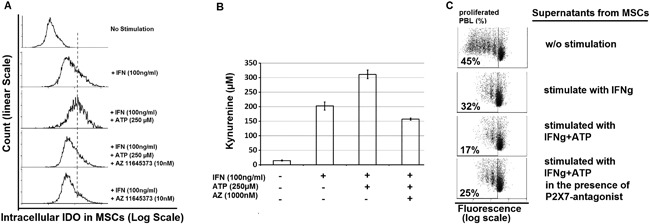
ATP promotes immunosuppressive MSCs by acting through P2 × 7‐Receptor. Bone marrow derived MSCs were cultured for four days in tryptophan saturated media in the presence of IFNγ alone or in combination with ATP ± P2 × 7 receptor antagonist (AZ11645373). MSCs were fixed, permeabilized and assessed for intracellular IDO expression by FACS (A), while culturing supernatant was analyzed for kynurenine (B), or used as conditioned media for culturing fluorenscent‐labelled PBL stimulated with anti‐CD3/anti‐CD28‐beads to induce their proliferation within 4 days (C). Shown are results from one representative out of four independent experiments

Consistent with our results on kynurenine production, we confirmed the expression of kynurenine producing enzyme (IDO) in MSCs by flow cytometry, showing enhanced expression of IDO in MSCs stimulated with ATP plus IFNγ compared to non‐stimulated MSC or MSCs stimulated with IFN alone (Figure 2A).

### 3.2 ATP effect on immunosuppressive MSCs is P2 × dependent

P2 × 7 is an ATP specific receptor which can be competitively inhibited by AZ11645373.

Bone marrow derived MSCs were cultured for 4 days in tryptophan saturated media in the presence of IFNγ alone or in combination with ATP ± P2 × 7 receptor antagonist (AZ11645373). MSCs were fixed, permeabilized and assessed for intracellular IDO expression by FACS (Figure 2A), while culturing supernatant was analyzed for kynurenine (Figure 2B), or used as conditioned media for culturing PBL stimulated with anti‐CD3/anti‐CD28‐beads to induce their proliferation. By culturing MSCs in the presence of this inhibitor, we could abolish the stimulatory effect of ATP on MSCs in terms of IDO expression (Figure 2A), kynurenine production (Figure 2B), and inhibition of lymphocyte proliferation (Figure 2C).

### 3.3 ATP enhances IFNγ‐induced inhibition of lymphocyte proliferation by MSCs

**Figure 3:**
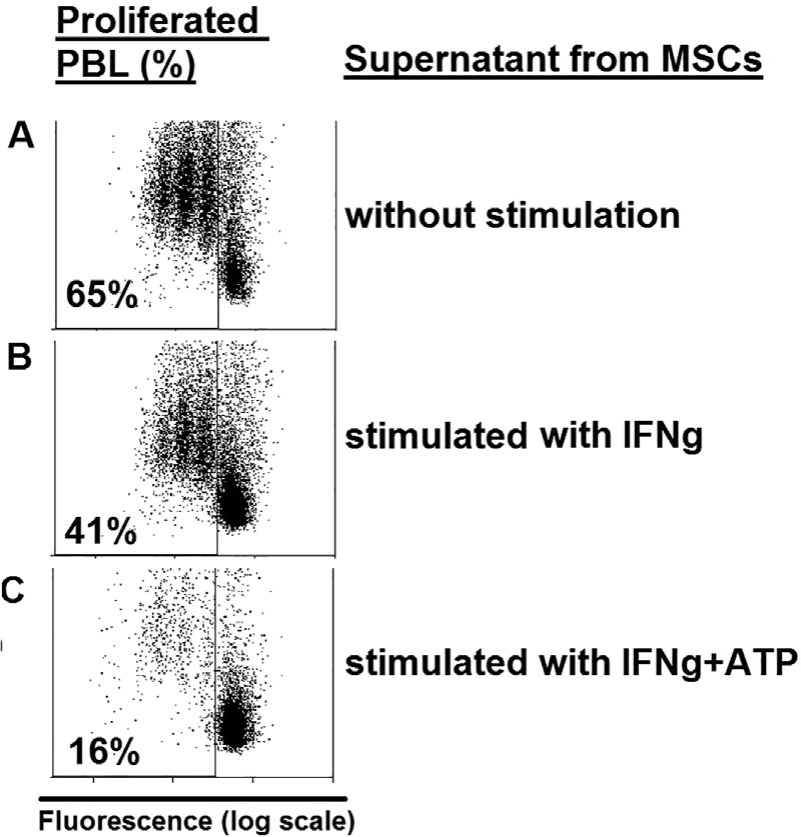
ATP enhances IFNγ‐induced inhibition of lymphocyte proliferation by MSCs. Bone marrow derived MSCs were cultured for four days in tryptophan saturated media in the presence of IFNγ (100 ng/mL) alone or in combination with ATP (250 μM). Thus obtained conditioned media was used to culture PBL in the presence of anti‐CD3/anti‐CD28‐beads (1 bead per 12.5 PBLs), Lymphocyte proliferation after 4 days was assessed by performing flow cytometry. Shown is the ratio of proliferating lymphocytes from one representative out of at least three independent experiments

Considering the fact that increased concentrations of kynurenine has an anti‐proliferative effect on lymphocytes, we aimed to assess the immunosuppressive impact of above shown enhanced kynurenine production, in the presence of ATP. Thus, we conducted proliferation assays with lymphocytes stimulated with anti‐CD3/anti‐CD28 beads in the presence of supernatants from MSCs which have been exposed for 4 days to IFNγ alone or to IFNγ in combination with ATP, and could confirm that ATP enhances IFNγ‐induced suppression of lymphocyte proliferation by MSCs (Figure 3). About 65% of PBLs proliferated following stimulation with anti‐CD3/anti‐CD28 beads (Figure 3A). This proliferation could be reduced to 41% in the presence of conditioned medium from IFNγ‐stimulated MSCs (Figure 3B). Compared to stimulation with IFNγ alone, MSCs stimulated with both IFNγ and ATP showed a significantly higher antiproliferative effect on PBL leading to further reduction of PBL proliferation by more than 60% (from 41% to 16%) (Figure 3C).

**Figure 4:**
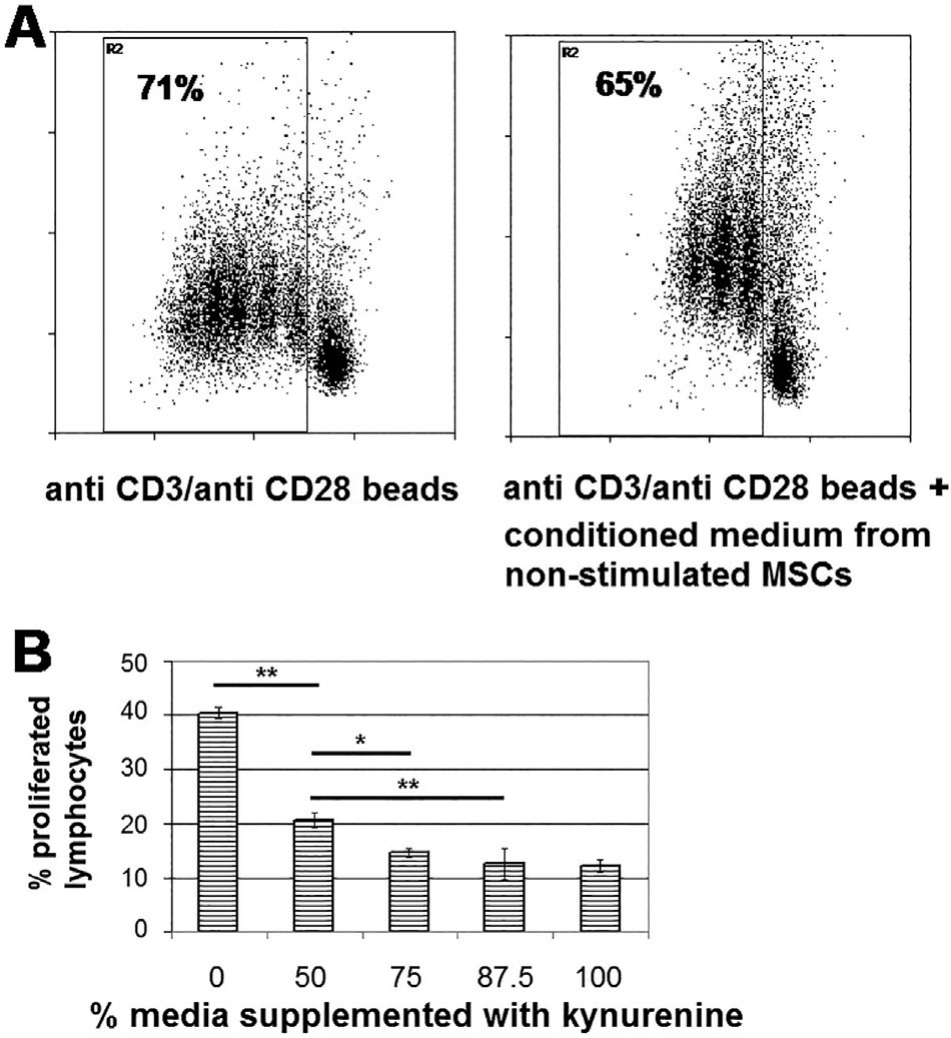
Kynurenine plays an important role in MSC‐induced inhibition of lymphocyte proliferation. (A) In the presence of anti‐CD3/anti‐CD28‐beads, CFSE‐labelled lymphocytes were incubated for 4 days in fresh media or conditioned media obtained from non‐stimulated MSCs cultured in media containing 100 μg/mL tryptophan. Lymphocyte proliferation was assessed by performing flow cytometry (FACS). Shown is the ratio of proliferated lymphocytes. Conditioned media from non‐stimulated MSCs does not inhibit lymphocyte proliferation. Shown are results from one representative out of at least three independent experiments. (B) Culturing media was supplemented with 200 μM kynurenine and added at indicated ratios to conditioned media from non‐stimulated MSCs, in order to mimic kynurenine production by stimulated MSCs. PBL were resuspended in above described kynurenine‐supplemented conditioned media and stimulated by anti‐CD3/anti‐CD28‐beads to induce proliferation. Kynurenine dose‐dependently inhibits PBL proliferation within 4 days. Shown is one representative experiment out of two independent experiments with asterisks indicating *P* values (*P ≤ 0.05 and **P ≤ 0.01)

**Figure 5:**
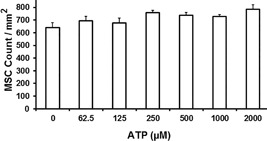
ATP does not influence MSC Proliferation. Bone marrow derived MSCs were cultured in the presence of indicated concentration of ATP for 4 days. Shown are results (mean ± standard deviation) form one representative out of seven independent experiments

The antiproliferative effect of conditioned media from MSCs could only be demonstrated following stimulation of MSCs with IFNγ ± ATP (Figure 3) and was not detectable when using supernatants from non‐stimulated MSCs (Figure 4A), thus excluding the possibility that inhibition of PBL proliferation may have been due to culturing PBL a in media deprived of nutrients by MSCs. To further exclude the possibility of nutrient deprivation by MSCs, we assessed MSC proliferation in the presence of ATP, and could show that ATP does not impact MSC proliferation and thus does not have any substantial influence on the consumption of nutrient within the culturing media (Figure 5).

In order to confirm that kynurenine is a crucial factor within the conditioned media responsible for inhibition of lymphocyte proliferation, we supplemented conditioned media from MSCs with graded concentration of kynurenine and could demonstrate a dose‐dependent antiproliferative effect of kynurenine on PBL (Figure 4B).

Remaining ATP within conditioned media by itself may influence lymphocyte proliferation, thus we excluded this possibility by demonstrating the absence of ATP within the conditioned media which we used for PBL proliferation assays (suppl. Figure S1).

## 4 DISCUSSION

Despite prevailing inflammation within the tumor microenvironment in vivo, and the possibility to generate potent tumor specific lymphocytes ex vivo, the eradication of tumor by host's immune response or by adoptively transferred antigen‐specific T cells is rarely successful, emphasizing the crucial impact of factors present within the tumor microenvironment and influencing immune response to tumor. Thus, gaining new insights into tumor microenvironment and targeting specific factors within tumor's micromilieu has become an increasing field of interest, in recent years.

With an improved understanding of tumor‐induced immunosuppression, we could develop novel therapies while exploiting a wide range of potential approaches such as restoring antitumor immune responses or eliminating tumor escape mechanisms, which are regarded as major problems in cancer therapies.^34^, ^35^

It has been well demonstrated that MSCs are recruited to the site of tumor,^9^, ^11^, ^12^, ^13^, ^14^ playing a pivotal role by interfering with other immune cells present in the surrounding tumor tissue. For this reason, it is highly relevant to determine how MSCs exert their immunoregulatory functions.

Since striking evidence indicates that (MSC‐derived) IDO contributes to tumor immune escape,^15^, ^16^, ^17^ our study focussed on ATP‐enhanced IDO expression in MSCs and its impact on lymphocyte proliferation which was assessed by culturing PBL in conditioned media derived from MSCs stimulated with predominant factors within inflammatory necrotic tissue of tumor, that is, IFNγ as a prototype of inflammation‐associated cytokines plus ATP^36^, ^37^ as a crucial DAMPs family member which is found at thousand times higher concentrations in tumor tissue.^31^, ^32^, ^33^

We applied ATP concentrations which are found within (necrotic) tumor tissue, in order to mimic the in vivo situation. As demonstrated here, ATP‐stimulated MSCs were capable of reducing PBL proliferation by more than 60% (from 41% to 16%) compared to the condition without ATP (Figure 3), this effect was closely related to IDO expression and subsequent kynurenine production (Figure 2). Using apyrase and P2 × 7 receptor antagonists (Figure 2) we could show that the ATP effect on MSC was specific and not due to ATP breakdown products such as AMP or adenosine,^35^ taking into account that MSCs constitutively express both enzymes (CD39 and CD73) which are able to dephosphorylate and further degrade ATP.

In contrast to published data on cytotoxic effect of ATP, we demonstrated that ATP at concentrations up to 1000 μM did not affect MSC proliferation (Figure 5), giving these cells the privilege to migrate and survive in necrotic areas and influence the microenvironment there.

Data presented here have implications for both, cancer therapy and therapies focussing on acceleration of wound healing, because of the similarities within the microenvironment of tumor and healing tissues.

## CONFLICTS OF INTEREST

The authors declare no competing financial interests.

## Supporting information

Additional supporting information may be found online in the Supporting Information section at the end of the article.

**Figure S1**. Conditioned media used for PBL Proliferations Assays did not contain remaining ATP. Bone marrow derived MSCs were cultured without any stimulation or in the presence of ATP, IFNg, or ATP plus IFNg for 4 days. Supernatant from thus cultured MSCs were tested for ATP.
