# Supplementary figures and images for: ATP promotes immunosuppressive capacities of mesenchymal stromal cells by enhancing the expression of indoleamine dioxygenase
Source: Immun Inflamm Dis. 2018 Oct 10;6(4):448–55. doi: 10.1002/iid3.236 (PMC6247240; doi:10.1002/iid3.236)

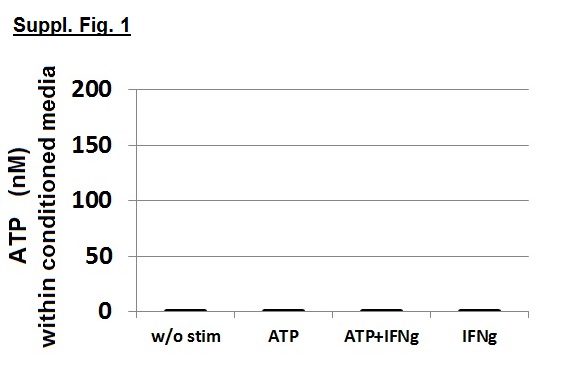

Supplement: Supplementary file 1 — Figure S1. Conditioned media used for PBL Proliferations Assays did not contain remaining ATP. Bone marrow derived MSCs were cultured without any stimulation or in the presence of ATP, IFNg, or ATP plus IFNg for 4 days. Supernatant from thus cultured MSCs were tested for ATP. [file IID3-6-448-s001.jpg]
